# DExD‐Box Helicase 21 Enhances Myometrial Contractions Through Thrombospondin‐1‐Mediated Increase in Cell Adhesion

**DOI:** 10.1111/jcmm.70268

**Published:** 2024-12-17

**Authors:** Lina Chen, Yanmin Jiang, Xiaodi Wang, Lele Wang, Junjie Bao, Zi Lv, Xiaoyan Sha, Zheng Zheng, Yunshan Chen, Kaiyuan Ji, Huishu Liu

**Affiliations:** ^1^ Guangzhou Key Laboratory of Maternal‐Fetal Medicine, Institute of Reproductive Health and Perinatology, Guangzhou Women and Children's Medical Center Guangzhou Medical University Guangzhou Guangdong China; ^2^ School of Medicine South China University of Technology Guangzhou Guangdong China

**Keywords:** cell adhesion, DDX21, myometrial contractility, THBS1

## Abstract

During labour, the myometrium transitions from a quiescent to an actively contracting state, governed by changes in gene expression. Identifying the pivotal transcription regulators involved in these gene expression alterations offers a useful strategy for addressing abnormal myometrial contractions. This study determined that the transcriptional regulator DExD‐Box Helicase 21 (DDX21) is upregulated in human myometrial tissues and myometrial smooth muscle cells (hMSMCs) during labour. DDX21 enhances hMSMC contractility through a mechanism that involves binding to thrombospondin 1 (THBS1) mRNA, a cell adhesion molecule, and promoting its transcription and subsequent protein expression. This upregulation of THBS1 increases cellular adhesion, which is crucial for effective myometrial contraction and for contractile function. Consequently, the DDX21‐THBS1 pathway could be a potential target for modulating key functions required for effective myometrial contraction.

## 1 Introduction

The myometrium, the muscular layer of the uterine wall, initiates rhythmic and powerful contractions during labour. Myometrial contractions are crucial for successful delivery by helping dilate and efface the cervix, preparing the birth canal for the delivery of the baby and assist in the descent and positioning of the foetus. Following childbirth, myometrial contractions continue to expel the placenta and compress the uterine blood vessels to prevent postpartum haemorrhage. Additionally, these contractions facilitate the process of uterine involution, enabling the uterus to return to its pre‐pregnancy size and shape [1]. Therefore, effective myometrial contractions are essential for safe and efficient delivery, impacting every stage from labour onset through postpartum recovery.

Regulation of myometrial contractions, crucial for successful childbirth, still remains incompletely understood. While it is known that hormonal signalling, inflammation and mechanical stretch play critical roles in initiating and sustaining myometrial contractions [2], the precise molecular pathways and cellular mechanisms governing myometrial excitability and contractility are not yet fully understood. The regulation of gene expression in the myometrium significantly influences uterine contractions and, by extension, the entire labour process. Epigenetic modifications including DNA methylation and histone acetylation, modulate gene expression during labour [3]. Transcription factors such as NF‐κB [4, 5] and activator protein‐1 (AP‐1) [6, 7, 8] upregulate genes linked to inflammatory responses and contraction‐associated proteins, respectively, ensuring effective myometrial contractility. The precise coordination of these epigenetic and genetic controls enables timely and effective myometrial contractions at term.

The integrated analysis of multi‐omics data has significantly advanced our understanding of key biological pathways and genes responsible for phenotypic changes. Our previous RNA sequencing (RNA‐seq) [9] and single‐cell RNA‐seq (scRNA‐seq) [10] analyses of human myometrial samples demonstrated significant transcriptional changes in the labouring myometrium compared to those in a non‐labouring state. A notable finding from the multi‐omics data was the widespread detection and altered expression of the DEAD (Asp‐Glu‐Ala‐Asp) box helicase family members, which are known for its roles in regulating various processes involving RNA homeostasis or biogenesis and function, including transcription, splicing, ribosome biogenesis, and RNA transport, as well as translation initiation [11, 12]. Among the members of this family, DDX21 stood out prominently because of its substantial fold change in expression and high expression abundance during labour. Like other DEAD‐box helicases, DDX21 participates in the unwinding of RNA structures, thereby increasing transcriptional machinery accessibility to DNA and enhancing the transcription process [13, 14]. The marked upregulation of DDX21 in the labouring myometrium implies its substantial in modulating the gene expression landscape necessary for efficient myometrial contractions. Understanding the molecular and cellular mechanisms of the new regulator DDX21 that modulates myometrial contractions is essential for improving interventions for labour complications, including preterm labour and labour dystocia.

To elucidate the role of DDX21 as a potential transcriptional regulator in myometrial contraction, its expression was examined in human myometrial tissues during both labouring and non‐labouring states. Subsequently, we explored the influence of DDX21 on the contractility of human primary uterine smooth muscle cells by employing small interfering RNA (siRNA)‐mediated gene silencing. In addition, we attempted to identify the downstream gene targets of DDX21 through RNA immunoprecipitation sequencing (RIP‐seq) and investigate the regulatory mechanisms through which DDX21 modulates myometrial contractility.

## 2 Materials and Methods

### 2.1 Human Tissues Collection

Human myometrial samples were obtained from term non‐labour or term in‐labour women who underwent caesarean delivery at Guangzhou Women and Children's Medical. All the puerperae were singleton and nulliparous status, without any pregnancy complications (such as hypertension, eclampsia or gestational diabetes), placenta previa or uterine fibroids. Labour was defined as the presence of regular palpable contractions along with cervical dilation. Myometrial samples were extracted from the upper edge of the lower segment uterine incision following caesarean section immediately after foetal and placental delivery. Blood contamination from the tissue samples was promptly removed by rinsing the samples with phosphate‐buffered saline (PBS, Gibco, Grand Island, NY, USA), and any attached decidua or adipose tissue was carefully excised using surgical scissors. The samples were then submerged in RNAlater solution (Sigma, Merck KGaA, Darmstadt, Germany) and stored at −80°C for future analysis. This study was approved by the Ethics Committee of Guangzhou Women and Children Medical Center (No. 2023066A01), and all participants provided written informed consent.

### 2.2 Primary Human Myometrial Smooth Muscle Cells (hMSMCs) Culture

Myometrium was obtained from non‐labour pregnant women who were isolated, with all blood vessels, attached decidua and adipose tissue carefully removed. The myometrium was then dissected into uniform 5 mm^3^ fragments and cultured in Dulbecco's Modified Eagle Medium (DMEM, Gibco, Grand Island, NY, USA) supplemented containing 10% foetal bovine serum (FBS, Thermo Fisher, Waltham, MA, USA) and 1% penicillin–streptomycin (Gibco) under conditions of 5% CO2 at 37°C. Following incubation for 7–10 days, the smooth muscle cells were identified by immunofluorescence targeting α‐smooth muscle actin (α‐SMA).

### 2.3 Reverse Transcription Quantitative Polymerase Chain Reaction (RT‐qPCR)

Total RNA from hMSMCs or myometrium was extracted and purified using RNeasy Plus Mini Kit (QIAGEN, Valencia, CA) following the manufacturer's instructions. The RNA concentration was measured using Multiskan Go (Thermo Fisher, Waltham, MA, USA). mRNA was reverse transcribed using PrimeScript RT Master Mix (TaKaRa, Otsu, Shiga, Japan). qPCR was using TB Green Premix Ex Taq II (TaKaRa, Otsu, Shiga, Japan) and performed by StepOnePlus Real‐Time PCR System (Applied Biosystems). The sequence of primers is listed in Table S1. The qPCR conditions were as follows: 95°C for 10 min, 40 cycles at 95°C for 15 s and 60°C for 1 min. The relative expression levels of mRNA were normalised with glyceraldehyde 3‐phosphate dehydrogenase (GAPDH) and were calculated using the 2^−ΔΔ*CT*^ method.

### 2.4 Western Blot

Myometrial tissues were lysed on ice for 10 min using the RIPA lysis buffer (Beyotime) and homogenised via high‐speed cryogenic grinding, followed by centrifugation at 12,000 rpm at 4°C for 5 min, then collected the supernatant. hMSMCs were collected after lysed in RIPA lysis buffer (Beyotime) and centrifuged at 12,000 rpm at 4°C for 5 min, then collected the supernatant. The protein concentrations were measured using a bicinchoninic acid (BCA) protein assay kit (Thermo Fisher, Waltham, MA, USA). Proteins were separated using SDS polyacrylamide gel electrophoresis (PAGE) and then transferred to polyvinylidene difluoride (PVDF) membranes (Millipore, Merck KGaA, Darmstadt, Germany) and were then probed with primary antibodies (GAPDH, Affinity, AF7021; DDX21, Proteintech, 10528‐1‐AP; THBS1, Abcam, ab267388) and subsequently with secondary antibodies (HRP‐conjugated Affinipure Goat Anti‐Rabbit IgG [H + L]). The proteins were quantified and imaged using BioRad's ChemiDoc XRS^+^ System and Image Lab Software, respectively. The relative expression levels of the proteins were normalised to GAPDH.

### 2.5 Immunofluorescence

Paraffin‐embedded tissue sections were deparaffinised and rehydrated, followed by antigen retrieval using citrate buffer (pH 6.0) at 98°C for 10 min. The cells were fixed with 4% paraformaldehyde (PFA) for 30 min, and then permeabilized with 0.1% Triton X‐100 for 20 min. The following steps were the same as those for both tissue specimens and cells. Following incubation at 37°C for 1 h with 10% goat serum to block non‐specific binding sites, then subjected to overnight staining at 4°C with primary antibodies (α‐SMA, 1:200, Abcam, ab7817; DDX21, 1:200, Proteintech, 10528‐1‐AP), followed by secondary antibodies (Goat anti‐Rabbit IgG‐Alexa Fluor 488, 1:500, Abcam, ab150077; Goat anti‐Mouse IgG‐Alexa Fluor 647, 1:500, Abcam, ab150115). The coverslips were mounted with DAPI‐containing VECTASHIELD (G1226, Servicebio, Wuhan, China). The images were photographed using Leica DMi8 fluorescence microscope.

### 2.6 Cell Transfection and Infection

hMSMCs were plated and reached approximately 70%–80% confluence, the cells were transfected with small‐interfering RNA (siRNA) using Interferin reagent (INTERFERin, 409–10, Polyplus) according to the manufacturer's protocol. The siRNA sense sequences (5′‐3′) were as follows: si‐NC‐F, UUCUCCGAACGUGUCACGUdTdT; si‐NC‐R, ACGUGACACGUUCGGAGAAdTdT; si‐DDX21‐F, GAGGUCAAUUUGAACGCAUdTdT; si‐DDX21‐R, AUGCGUUCAAAUUGACCUCdTdT; si‐THBS1‐F, CGUGGUGUCUGUGGAAGAAdTdT; si‐THBS1‐R, UUCUUCCACAGACACCACGdTdT. The cells were collected for subsequent experiments after 24–72 h of infection.

For overexpression studies, hMSMCs were infected with DDX21 overexpression lentiviral particles (oe‐DDX21) at a multiplicity of infection (MOI) of 50. DDX21 overexpression lentivirus was constructed using lentiviral vector pCDH‐CMV‐MCS‐EF1‐copGFP‐T2A‐Puro and Homo‐DDX21 coding DNA sequence (CDS). Cells were collected for subsequent experiments after 24–72 h of infection.

### 2.7 Cell Contractility Assays

The contractility of hMSMCs was assessed using a cell contraction assay kit (Cell Biolabs Inc., San Diego, CA, USA) following the manufacturer's instructions. Initially, the cells were trypsinised and suspended, and a collagen solution was prepared by diluting it with DMEM (1.0 mg/mL). Subsequently, the cells and collagen solution were thoroughly mixed in individual wells of a 24‐well plate (1.5 × 10^5^ cells/well). This mixture was allowed to gel by incubating it at 37°C for 1 h. Following gel formation, 1.0 mL of DMEM containing 10% FBS was introduced into each well. After a 24 h culture period, the gels were released. To monitor the contractile activity, the gels were captured using the ChemiDoc XRS+ system, and the area of the gel was measured using ImageLab software. A reduction in the area of the floating gel indicates an increase in baseline contractile tension.

### 2.8 RIP Experiment, Library Preparation and Sequencing

hMSMCs were plated and confluence reached approximately 90% (5 × 10^7^ cells), the cells were rinsed with cold PBS and then scraped off. The cells were subsequently collected by centrifugation. The cells were resuspended in RIP lysis buffer containing a protease inhibitor cocktail and RNase inhibitor, then incubated on ice for 15 min, and centrifuged at 1600 rpm at 4°C for 10 min to collect the supernatant. Magnetic beads pre‐incubated with either DDX21 (Proteintech, 10528‐1‐AP) or IgG (Proteintech, 10284‐1‐AP) antibodies were then combined with RIP immunoprecipitation buffer containing ethylene diamine tetraacetic acid (EDTA), RNase Inhibitor and the cell supernatant. The bead‐antibody complexes and cell lysates underwent rotation at 4°C overnight. Subsequently, the beads were washed four times with RIP wash buffer. The supernatant was then separated from the magnetic beads and the RNA was extracted using TRIzol reagent (Invitrogen).

The stranded RNA sequencing library was constructed by using the KC‐DigitalTM Stranded mRNA Library Prep Kit for Illumina (Catalogue No. DR08502, Wuhan Seqhealth Co. Ltd., China). The kit eliminates duplication bias in PCR and sequencing steps by using a unique molecular identifier (UMI) of eight random bases to label the pre‐amplified cDNA molecules. The library products corresponding to 200–500 bps were enriched, quantified, and finally sequenced on the DNBSEQ‐T7 sequencer (MGI Tech Co. Ltd., China) with the PE150 model.

### 2.9 RIP‐Seq Data Analysis

Raw sequencing data was first filtered by Trimmomatic (v 0.36). Clean Reads were further treated with in‐house scripts to eliminate duplication bias introduced in library preparation and sequencing. In brief, clean reads were first clustered according to the UMI sequences, in which reads with the same UMI sequence were grouped into the same cluster. Reads in the same cluster were then compared to each other by pairwise alignment, while reads with a sequence identity of over 95% were extracted to a new sub‐cluster. After all sub‐clusters were generated, multiple sequence alignment was performed to get one consensus sequence for each sub‐clusters. After these steps, any errors and biases introduced by PCR amplification or sequencing were eliminated.

The de‐duplicated consensus sequences were used for protein binding site analysis. They were mapped to the human genome ( *H. sapiens* , GRCh38) using hisat2 with the default parameters. The macs2 callpeak was used for peak calling. The peaks annotation and peaks distribution were analysed using ChIPseeker package by R (version 4.3.3). Sequence motifs enriched in peak regions were identified using Homer (version 4.10). Kyoto encyclopaedia of genes and genomes (KEGG) and Reactome pathway enrichment analysis was implemented by DAVID (version 6.8) [15] with a *p* value cut‐off of 0.05 to judge statistically significant enrichment pathways.

### 2.10 Real‐Time Cell Adhesion Assay

An E‐Plate 16 was coated 50 μL of fibronectin working solution (10 μg/mL) and incubated in a CO_2_ incubator for 30 min, then removed the fibronectin. Subsequently, 50 μL of DMEM medium and 100 μL of hMSMCs suspension (5000 cells) were added to each well. The E‐Plate 16 was immediately placed in the incubator on the xCELLigence RTCA Instrument (Agilent Technologies, Santa Clara, CA, USA), which was set to measure every 10 min continuously for 6 h. This instrument utilises microelectronic biosensors to assess cell adhesion to the well's bottom and translates the signals into a relative cell index. RTCA provides quantitative insights into the dynamic changes in the strength of cell adhesion over time.

### 2.11 Statistical Analysis

Data were calculated using GraphPad Prism 8.4.0 software (San Diego, CA, USA), The results were expressed as means ± SEM. Group comparisons were analysed using Student's *t*‐test and one‐way ANOVA test for two and multiple groups, respectively. The correlation between two mRNA expression levels was analysed using a two‐tailed Pearson correlation. The difference in cell adhesion over time was analysed using two‐way ANOVA, with Tukey's multiple comparisons test. *p* < 0.05 was considered statistically significant.

## 3 Results

### 3.1 DDX21 Was Upregulated in Labouring Myometrial Tissues and hMSMCs

**FIGURE 1:**
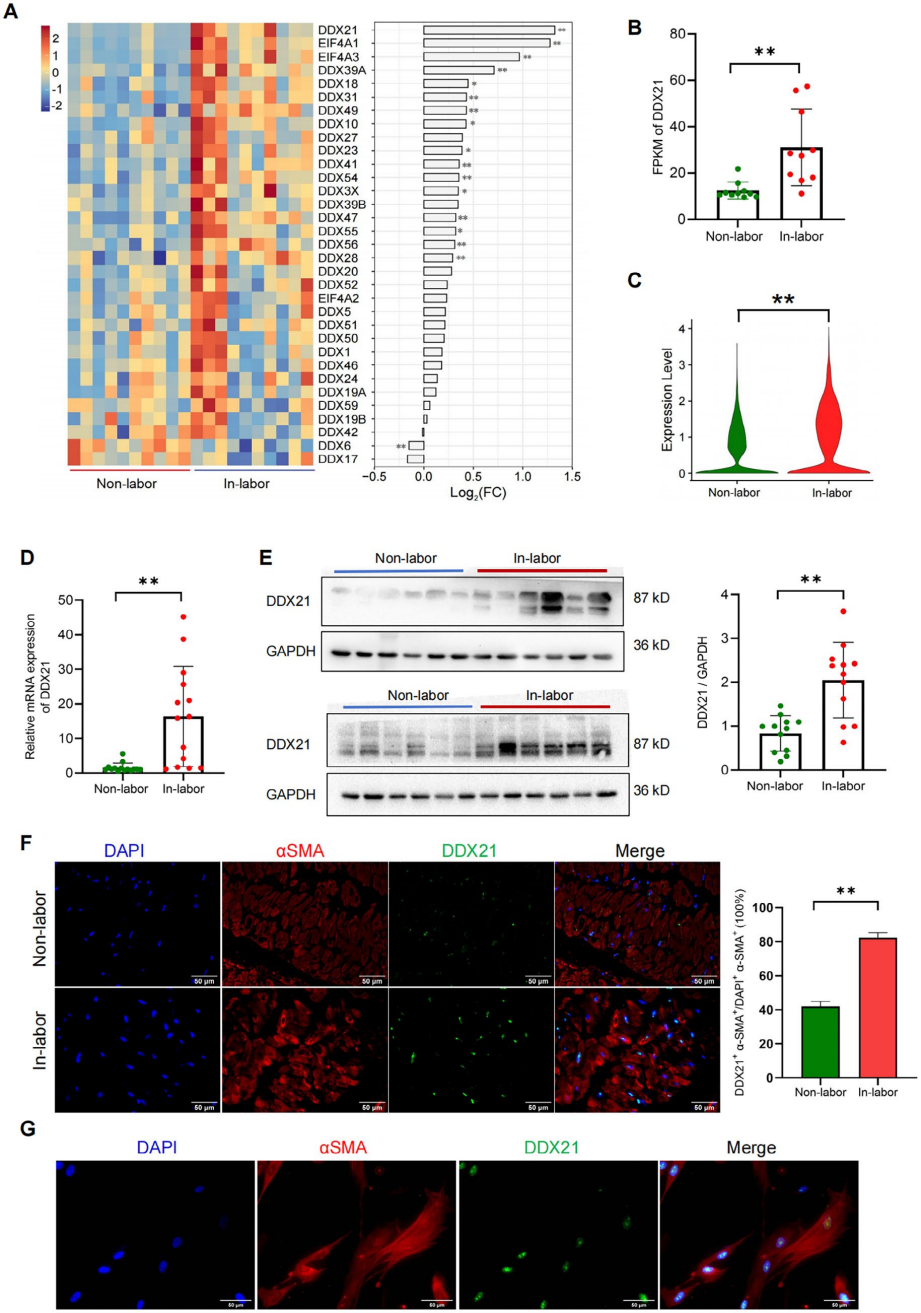
Omics‐based analysis was conducted to screen DDX21 as a regulator of myometrial contraction. (A) Heatmap and bar graph depicting the expression of DEAD‐box RNA helicase family members in myometrium (RNA‐seq). (B) Expression of DDX21 mRNA in myometrium (RNA‐seq). (C) Expression of DDX21 mRNA in uterine smooth muscle cells (scRNA‐seq). (D) RT‐qPCR analysis of DDX21 mRNA levels in myometrium. (E) Western blot assessment of DDX21 protein expression in myometrium. (F, G) Immunofluorescence visualisation of DDX21 expression in myometrium (F) and hMSMCs (G). **p < 0.01. RNA‐seq data reported in GEO repositories, with accession number GSE181348; scRNA‐seq data reported in GSA repositories, with accession number HRA002852.

In our previous study, we conducted comprehensive analyses of both global and single‐cell transcriptomes of human myometrial samples from full‐term pregnant women during labour (in‐labour) and not in labour (non‐labour) states, aiming to elucidate potential molecular mechanisms that critically influence the dynamics of uterine contractions during labour [9, 10]. The results showed significant differences in gene transcription levels between the quiescent and contracted states of the myometrium. Notably, the members of DEAD‐box RNA helicase family were extensively detected. DDX21, in particular, exhibited a significant upregulation in the in‐labour myometrium (log_2_ FC = 1.323, *p* = 0.006), showing the highest fold change and high expression abundance (Figure 1A,B). Furthermore, single‐cell profiling further highlighted the higher level of DDX21 in hMSMCs within the labouring group relative to the non‐labour group (Figure 1C). These omics‐based findings indicate that RNA helicases contribute to regulating uterine contractions during labour, with DDX21 identified as a potential key factor.

To validate the above transcriptomic results, the expression levels of DDX21 mRNA and protein in myometrium, along with the cellular localization of DDX21, were verified in clinical myometrium samples. The findings indicated significant upregulation of DDX21 in in‐labour myometrium (Figure 1D–F), which was consistent with the transcriptomic data. Nuclear localisation of DDX21 in hMSMCs (Figure 1G) further supported its role in transcriptional regulation.

### 3.2 DDX21 Contributed to Myometrial Contraction

**FIGURE 2:**
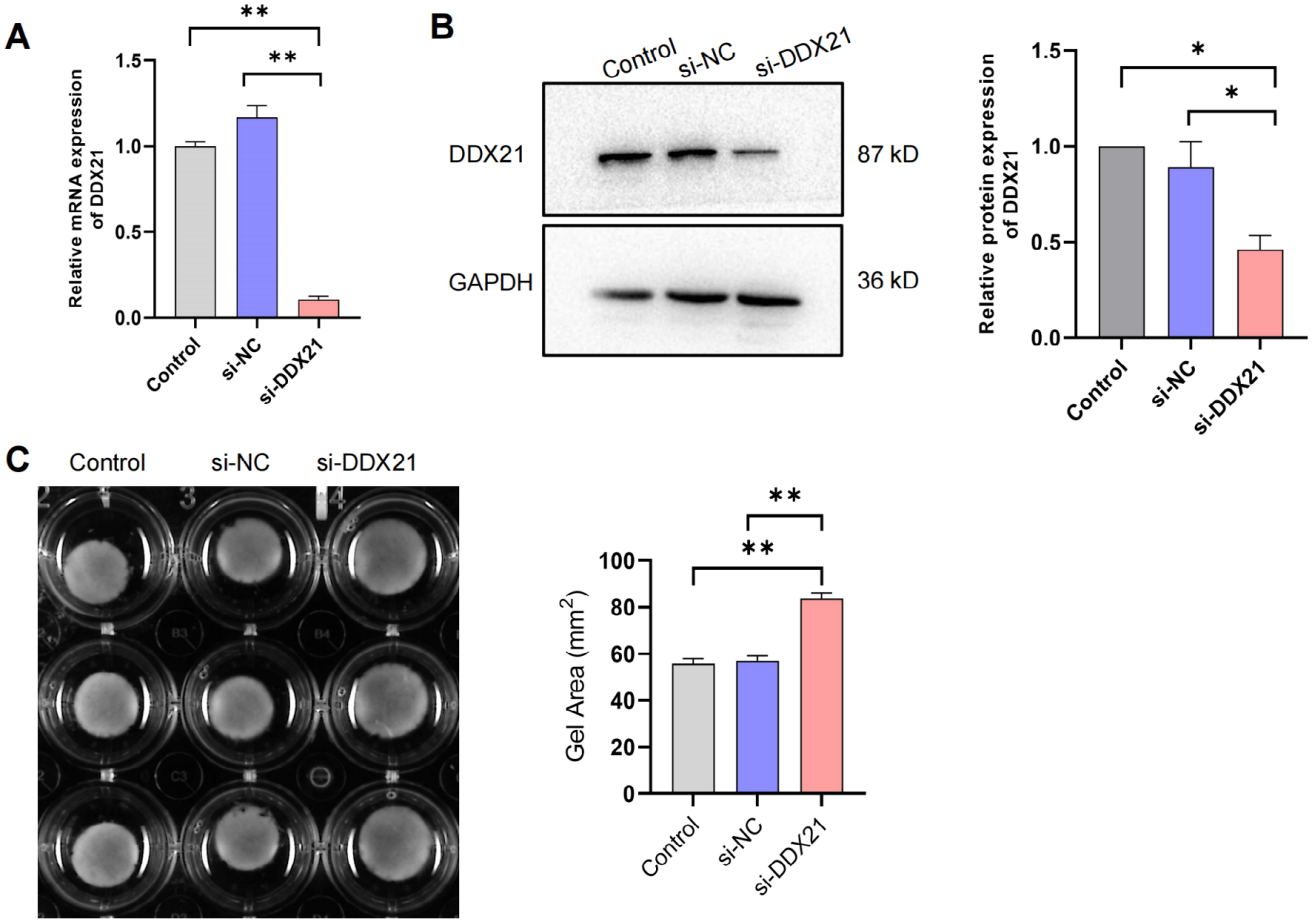
Impact of DDX21 silencing on contractile function in hMSMCs. (A, B) RT‐qPCR (A) and western blot (B) analyses assessing of mRNA and protein expression levels following si‐DDX21 transfection. (C) Collagen gel contraction assay evaluating the contractile capacity of cells transfected with si‐DDX21. *p < 0.05, **p < 0.01.

The function of DDX21 in myometrial contraction was further investigated by transfecting primary hMSMCs with siRNA. Subsequent RT‐qPCR and western blot analyses revealed a significant reduction in both mRNA and protein expression levels of DDX21 (Figure 2A,B). The collagen gel contraction assay showed that inhibition of DDX21 expression resulted in a larger gel area compared to the control group (Figure 2C), indicating reduced cell contraction ability.

### 3.3 Cell Adhesion Molecule THBS1 Was the Downstream Target Gene of DDX21

**FIGURE 3:**
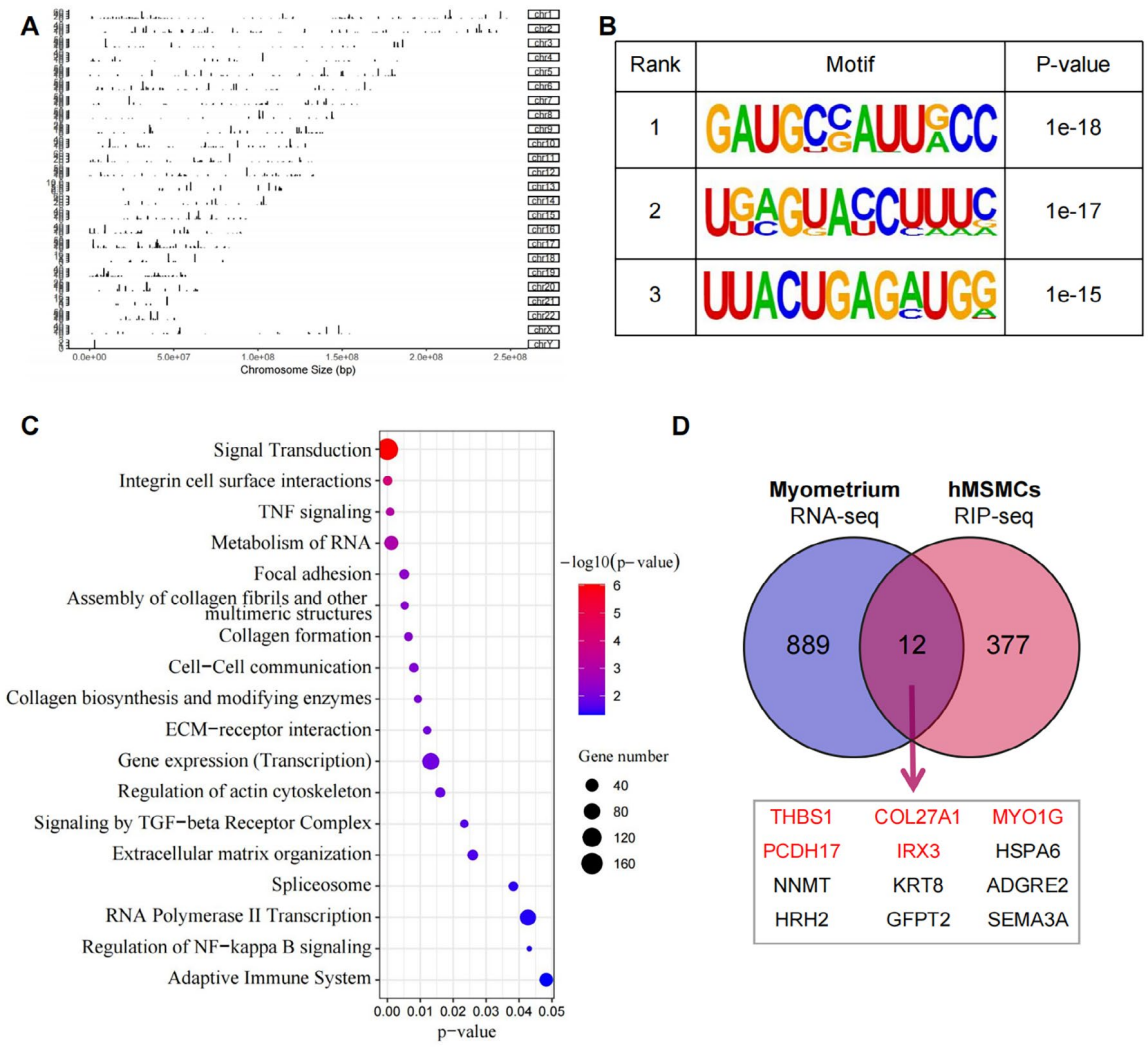
Analysis of putative downstream target genes of DDX21 with RIP‐seq in hMSMCs. (A) Distribution of DDX21 RIP‐seq peaks across the human genome. (B) Motifs identified at DDX21‐binding sites. (C) Pathway enrichment analysis of DDX21‐binding mRNAs. (D) Overlap of mRNAs upregulated in labouring myometrium (RNA‐seq) with those bound by DDX21 in hMSMCs (RIP‐seq).

**FIGURE 4:**
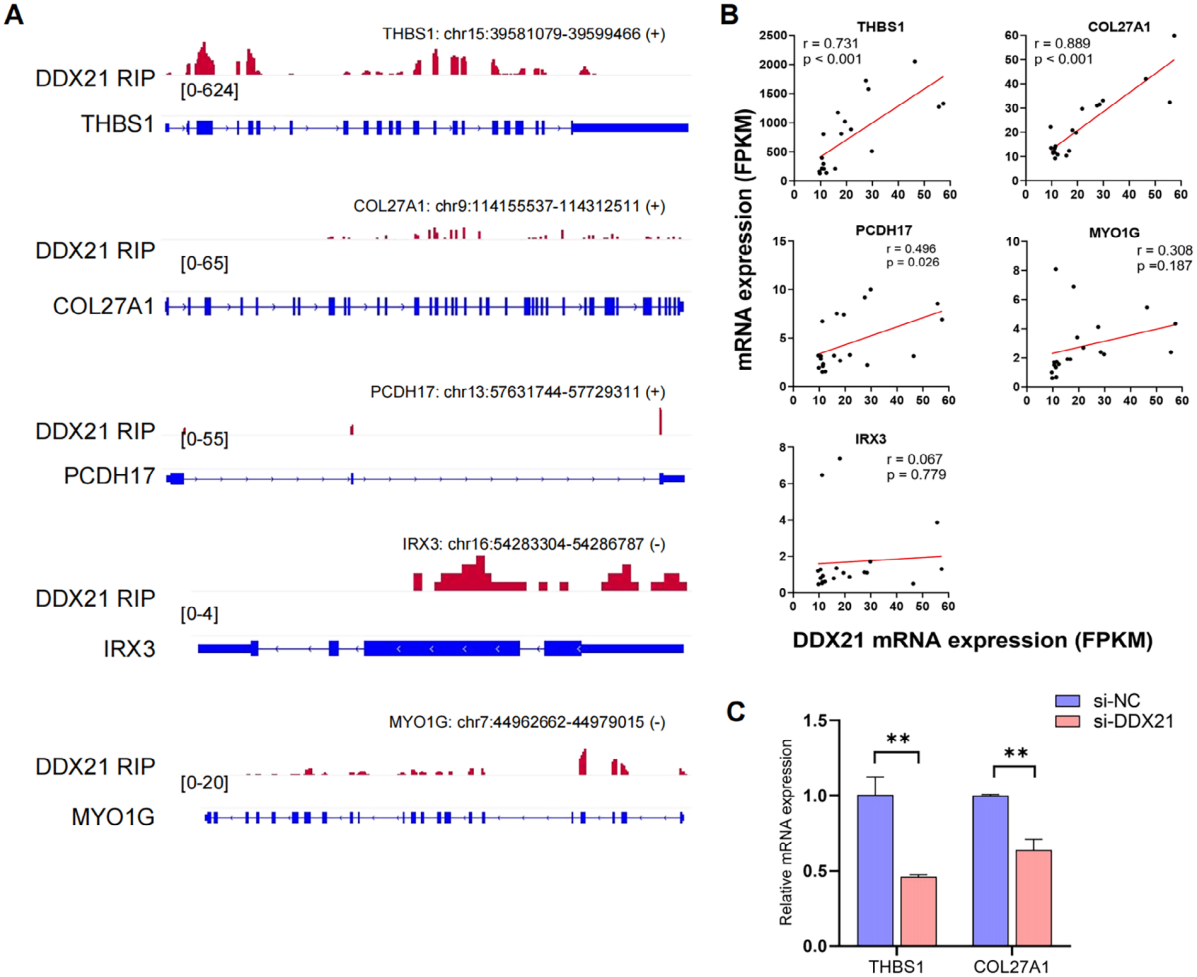
THBS1 was identified as a downstream target gene of DDX21. (A) Mapping of DDX21 RIP‐seq peaks among candidate target genes. (B) Correlation between mRNA expression of DDX 21 and candidate target genes in myometrium (RNA‐seq). (C) Alterations in mRNA levels of the candidate target gene after DDX21 knockdown in hMSMCs. **p < 0.01.

RIP‐seq was conducted to identify the downstream target genes regulated by DDX21 in hMSMCs. Based on the global analysis of the RIP‐seq data, a total of 1,087 peaks were identified after excluding those located in the distant intergenic region (Table S1). These peaks were mapped to the human reference genome NCBI GRCh38, identifying 901 mRNAs, with the highest peak density located on chromosome 1 (Figure 3A). Figure 3B lists the top three motif sequences enriched within DDX21 peaks. The signal pathway enrichment analysis of the genes significantly bound by DDX21 (*p* < 0.05) revealed an enrichment of genes involved in focal adhesion, TNF signalling, NF‐kB signalling and adaptive immune system, which are related to myometrium contraction. Additional enrichment was also observed in pathways involved in signal transduction, RNA metabolism and mRNA splicing. Notably, several pathways associated with cell adhesion were identified, such as collagen formation, regulation of actin cytoskeleton and extracellular matrix organisation (Figure 3C). To further narrow down the potential downstream target genes of DDX21, we overlapped the genes bound by DDX21 with those upregulated (fold change > 2) in the in‐labour myometrium. Consequently, we identified 12 candidate target genes, of which five (THBS1 [16], MYO1G [17], PCDH17 [18], IRX3 [19] and COL27A1 [20]) are associated with cell adhesion and connectivity functions (Figure 3D). An analysis of the peak signals of these candidate target genes revealed that DDX21 exhibits strong peak signals at THBS1 and COL27A1 (Figure 4A). Investigating the mRNA expression correlation between these candidate targets and DDX21 according to the myometrium RNA‐seq data. The results showed that THBS1 and COL27A1 were positively correlated with the expression of DDX21 (*p* < 0.001) (Figure 4B). Following DDX21 knockdown in hMSMCs, both THBS1 and COL27A1 expressions significantly decreased, with THBS1 showing a more pronounced fold change (Figure 4C). Based on these results, it is evident that THBS1 mRNA can be bound by DDX21, and its transcription level is regulated by DDX21, indicating THBS1 as a highly credible downstream target of DDX21.

### 3.4 DDX21 Enhances Cell Adhesion and Contractility via THBS1

**FIGURE 5:**
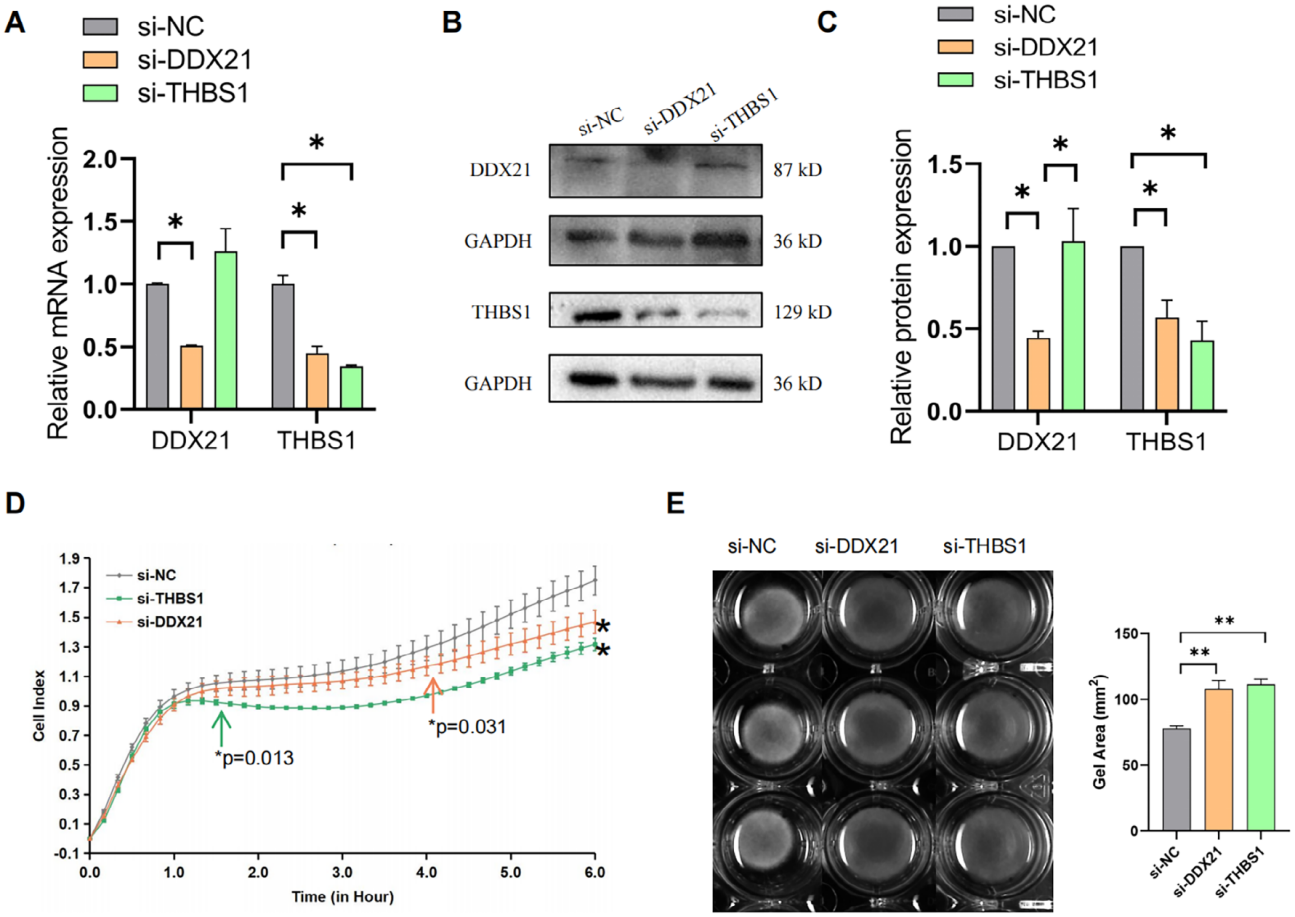
Inhibition of DDX21 or THBS1 on adhesion and contraction function of hMSMCs. (A–C) RT‐qPCR (A) and western blot (B, C) analyses assessing mRNA and protein expression levels of DDX21 and THBS1 in cells transfected with si‐DDX21 or si‐THBS1. (D) RCTA assay was used to detect the adhesion of cells transfected with si‐DDX21 or si‐THBS1. (E) A gel contraction test was used to detect the contraction function of cells transfected with si‐DDX21 or si‐THBS1. *p < 0.05, compared with si‐NC. *p < 0.05, **p < 0.01.

**FIGURE 6:**
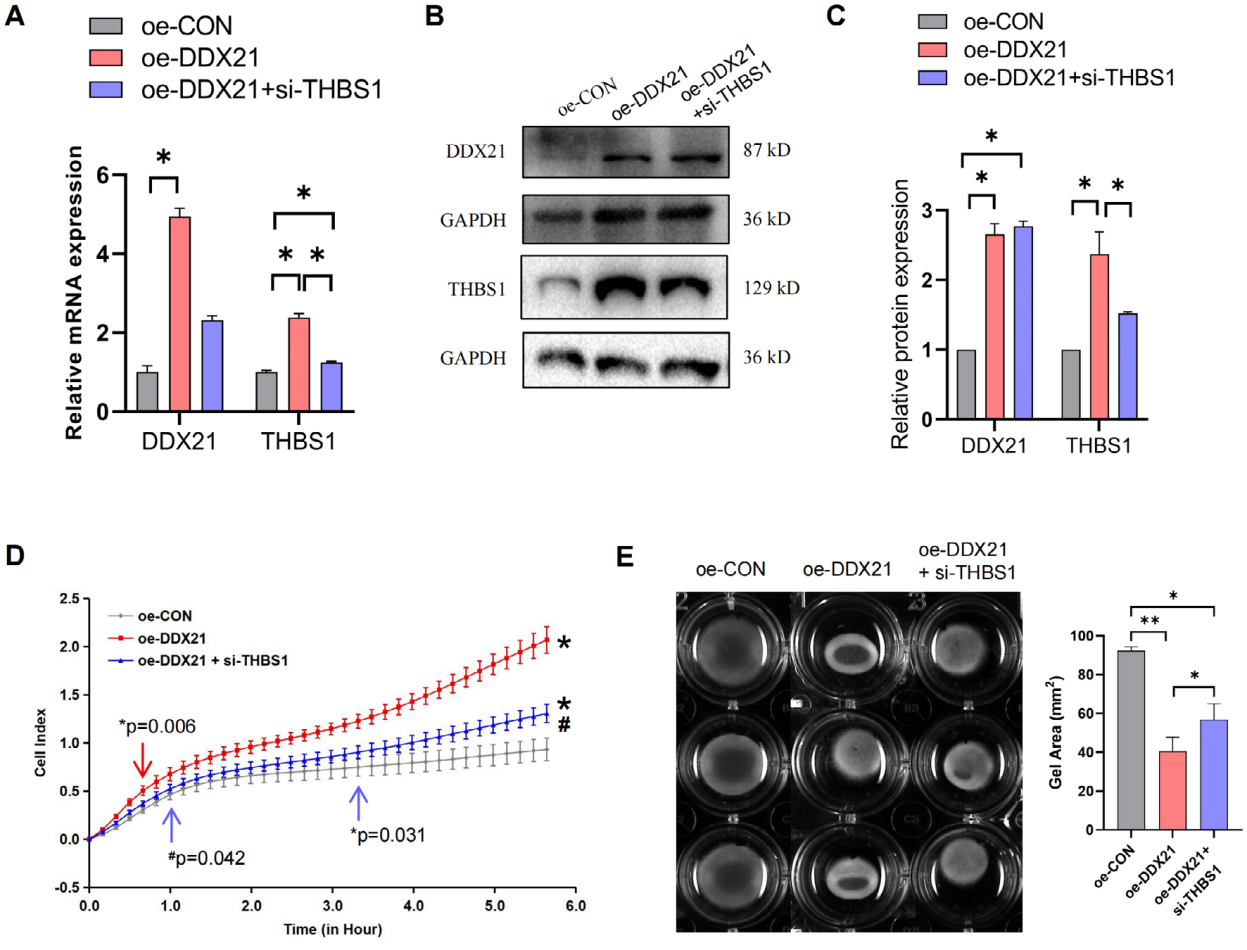
Overexpression of DDX21 on adhesion and contraction function of hMSMCs. (A–C) RT‐qPCR (A) and western blot (B, C) analyses assessing mRNA and protein expression levels of DDX21 and THBS1 in cells overexpression of DDX21 or simultaneous overexpression of DDX21 and inhibition of THBS1 expression. (D) RCTA assay was used to detect the cell adhesion ability after overexpression of DDX21 or simultaneous overexpression of DDX21 and inhibition of THBS1 expression. (E) Gel contraction test was used to detect the contraction function of cells after overexpression of DDX21 or simultaneous overexpression of DDX21 and inhibition of THBS1 expression.*p < 0.05, compared with oe‐CON; #*p* < 0.05, compared with oe‐DDX21. *p < 0.05, **p < 0.01.

**FIGURE 7:**
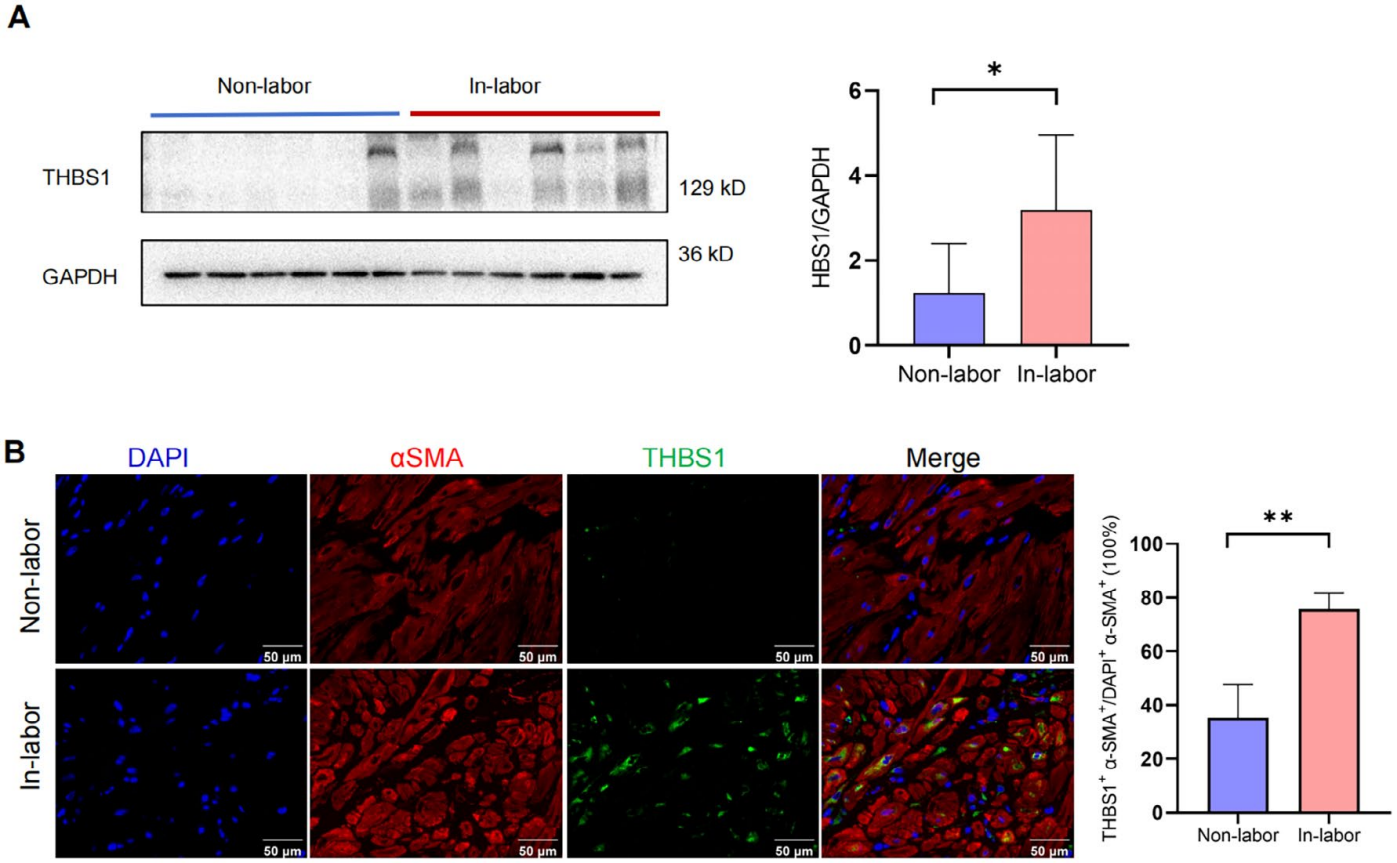
THBS1 was highly expressed in the non‐labour myometrium. (A, B) Western blot (A) and immunofluorescence (B) analyses of THBS1 protein expression in human non‐labour and in‐labour myometrium. *p < 0.05, **p < 0.01.

Myometrial contraction relies on the effective coordination interaction of hMSMCs, which require strong adhesion among cells and to the extracellular matrix to function effectively [21]. THBS1 is recognised as a pivotal cell adhesion molecule [16, 22]. To further elucidate the influence of DDX21 on THBS1‐mediated contraction of hMSMCs, DDX21 or THBS1 expression was inhibited in hMSMCs to decipher their effects on cellular adhesion and contractility using RTCA and collagen gel contraction assays, respectively. The results indicate a significant reduction in THBS1 expression at both mRNA and protein levels following the suppression of DDX21 or THBS1 (Figure 5A–C). The cell adhesion and contraction capabilities also weakened compared to the control group (Figure 5D,E). Conversely, overexpression of DDX21 enhanced the expression of THBS1 (Figure 6A–C), which subsequently increased cell adhesion and contraction in hMSMCs (Figure 6D,E), indicating a positive regulatory effect of DDX21 on hMSMC functions. Moreover, simultaneous inhibition of THBS1 expression partially reversed the enhancements induced by DDX21 overexpression (Figure 6A–E), delineating their combined effects on cellular adhesion and contractility. These results suggest a synergistic interaction between DDX21 and THBS1 in regulating hMSMCs function. The physiological relevance of this finding is further supported by increased THBS1 protein levels in the in‐labour myometrium relative to the non‐labour group (Figure 7A,B).

## 4 Discussion

The myometrium transitions from a quiescent state to a contracting state during labour, accompanied by changes in gene transcription and protein expression [23]. These molecular changes trigger alterations in the biological functions and signalling pathways of the myometrium, which we demonstrated in our previous multi‐omics studies [9, 10]. Current studies tend to primarily focus on identifying and validating the differences in gene expression related to uterine contraction. However, there has been relatively less exploration into the complex gene regulatory networks and their underlying mechanisms. Effective transcriptional regulators are promising targets for controlling myometrial contractions. In this study, we identified the RNA helicase DDX21 as a target based on transcriptomic data, revealing its elevated expression during the contractile state in human myometrial tissues and cells. Cellular studies elucidated the role of DDX21 in enhancing cell contractility, a process mediated by upregulating its downstream target gene THBS1, which in turn enhances cell adhesion and contraction capabilities. These results highlight the potential of DDX21 as a critical mediator in the regulation of myometrial function.

The RNA helicase DDX21 is a central molecule in RNA metabolism and relies on ATP hydrolysis to catalyse the unwinding of RNA structures, separation of RNA double strands and restructuring of RNA–protein complexes. Previous studies have highlighted its critical role in transcriptional regulation, particularly during transcriptional elongation, where it facilitates the progression of RNA polymerase movement along the DNA template by unwinding DNA–RNA hybrids [13, 14]. DDX21 has been implicated in the development of various cancers, including breast [24], gastric [25], colorectal [26] and melanoma [27] cancers. Despite these associations, its role in parturition remains unexplored. Our study results confirm that DDX21 acts as a positive contractile regulator in primary hMSMCs.

The RIP‐seq data indicate that DDX21 binds to numerous mRNAs, influencing multiple signalling pathways related to cell contraction, particularly those associated with cell adhesion. During labour, the cytoskeleton and morphology of hMSMCs undergo significant changes, which are dependent on cell adhesion to the extracellular matrix. Enhanced adhesion among hMSMCs allows for tighter and more organised connections, enabling them to contract in a coordinated manner as a unified system. This coordination facilitates the rapid transmission of contraction signals throughout the myometrium, thereby improving the overall effective and synchronised uterine contractions [28, 29, 30]. By integrating transcriptomics and experimental results, THBS1 was identified as a high‐confidence downstream target gene of DDX21. THBS1 is an adhesive glycoprotein that mediates both cell–cell and cell–matrix interactions, binding to various proteins, such as fibronectin, laminin, type V collagen and integrin α‐V/β‐1. This interaction not only facilitates the physical attachment of cells to the extracellular matrix but also triggers signalling pathways that influence cellular behaviours, such as migration, proliferation and in the context of myometrial cells, contraction [31, 32]. Our study shows that inhibiting THBS1 expression weakened cell adhesion to fibronectin and reduced contractile ability. These findings suggest that DDX21 may enhance cell adhesion by promoting THBS1 transcription, subsequently strengthening the myometrial contractility.

Preterm labour defined as labour ocurring between 28 and 37 weeks of gestation, involves complex factors that activate the ‘common pathway of labour’—cervical ripening, membrane‐decidual activation and uterine contractions—paralleling mechanisms seen in spontaneous term labour. To investigate the DDX21‐THBS1 axis in preterm labour, gene expression profiles from preterm spontaneous labour and non‐labour myometrial tissues were compared using the publicly available GSE134447 dataset [33]. Results indicated significant upregulation of both DDX21 and THBS1 in the preterm labour group, implying a role in promoting myometrial contraction, akin to its function in term labour. This observation is consistent with our primary findings on the role of the DDX21–THBS1 pathway in enhancing myometrial contraction and cell adhesion. Further investigation into this axis across the term and preterm labour may offer novel strategies for preterm labour management.

Our RIP‐seq data also indicated that DDX21 binds to c‐JUN and JUND mRNAs. Previous studies have shown that DDX21 can directly interact with the transcription factor c‐JUN, contributing to c‐JUN‐mediated activation of target gene transcription [24, 34]. As a member of AP‐1 transcription factor family, c‐JUN binds to the promoter region of the uterine contraction‐associated protein connexin 43 (CX43), thereby enhancing CX43 transcription [6, 7]. This interaction suggests a possible mechanism through which DDX21 may influence myometrial contractions by regulating c‐JUN expression, although additional research is still needed to validate this hypothesis.

DDX21 regulates transcription through its RNA helicase activity, involvement in chromatin remodelling and interactions with transcription factors and co‐regulators [13, 35, 36]. These diverse mechanisms underscore the critical role of DDX21 in maintaining proper cellular function and gene expression. The interaction between DDX21 and THBS1 mRNA in hMSMCs points to a regulatory mechanism where DDX21 may affect the stability and expression of THBS1 mRNA, thereby impacting cell adhesion and motility, which are crucial for remodelling myometrial contractions. The precise regulatory sites of DDX21 on THBS1 mRNA and the detailed regulatory mechanisms remain unclear. Further experimental studies are needed to delineate these molecular interactions and clarify how DDX21 regulates THBS1 and potentially other target mRNAs, providing deeper insights into its broader physiological roles.

Abnormal uterine contractions can lead to severe complications during delivery, adversely affecting both maternal and neonatal health. Excessively strong or frequent contractions can induce premature rupture of membranes and preterm labour [37, 38], while insufficient contractions may result in prolonged labour or obstructed labour [39]. In this study, we identified DDX21 as a novel transcriptional regulator that influences myometrial contraction through the regulation of THBS1. The interaction between DDX21 and THBS1 suggests a potential mechanism underlying the modulation of hMSMCs activity. Given the regulatory roles of both molecules in contraction dynamics, the DDX21–THBS1 axis represents a promising target for addressing uterine contractility issues and labour dysfunctions.

## Author Contributions

**Lina Chen:** conceptualization (equal), data curation (equal), investigation (equal), methodology (equal), writing – original draft (equal), writing – review and editing (equal). **Yanmin Jiang:** data curation (equal), formal analysis (equal), resources (equal). **Xiaodi Wang:** data curation (equal), formal analysis (equal), methodology (equal). **Lele Wang:** methodology (equal), resources (equal), validation (equal). **Junjie Bao:** methodology (equal), resources (equal), validation (equal). **Zi Lv:** data curation (equal), formal analysis (equal), resources (equal), software (equal). **Xiaoyan Sha:** formal analysis (equal), validation (equal), visualization (equal). **Zheng Zheng:** resources (equal), validation (equal), visualization (equal). **Yunshan Chen:** formal analysis (equal), resources (equal), writing – review and editing (equal). **Kaiyuan Ji:** conceptualization (equal), investigation (equal), supervision (equal), writing – review and editing (equal). **Huishu Liu:** funding acquisition (lead), supervision (equal), writing – review and editing (equal).

## Ethics Statement

This study was approved by the ethics committee of Guangzhou Women and Children Medical Center (No. 2023066A01).

## Conflicts of Interest

The authors declare no conflicts of interest.

## Supporting information


**Table S1.** Peaks of DDX21 RIP‐seq in hMSMCs.

## Data Availability

The data that support the findings of this study are openly available in GSA at https://ngdc.cncb.ac.cn, reference number (PRJCA010740).
